# Notes on Autopsies Made at Columbia Veterinary College and School of Comparative Medicine

**Published:** 1881-07

**Authors:** E. C. Wendt


### NOTES ON AUTOPSIES MADE AT COLUMBIA VETERINARY COLLEGE AND SCHOOL OF COMPARATIVE MEDICINE.

BY PROFESSOR E. C. WENDT.

V.—HEMORRHAGIC SPINAL MENINGITIS IN A MONKEY.—A small white-throated cebus (*Cebus hypolemus*) was sent to the anatomical department of the College by W. F. Conklin, Esq., Superintendent Central Park Menagerie. It was stated that the animal had been afflicted for the past six months with paralysis of the hind limbs. It was further added that this was a troublesome and rather frequent symptom in the monkeys at the Park.

At the autopsy, a fairly nourished body, without external marks of violence, was found. The organs of the chest were apparently quite healthy. The same was noted with reference to the abdominal organs. In opening the spinal canal, a striking picture of hemorrhagic meningitis was seen. The cervical and upper dorsal regions showed only a few scattered patches of hemorrhagic exudation, but, lower down, and especially at the dorso-lumbar region, the meninges were obscured by numerous irregular patches of a bloody fibrinous material. On removal of the dura-mater, which was adherent at many points, a few sanguineous patches were also found over the surface of the pia-mater. The substance of the cord itself was slightly congested, the hyperæmia being most evident in the gray substance. In the brain, or its meningeal coverings, inflammatory foci, or blood patches, were not found.

